# Responder rates with eptinezumab over 24 weeks in patients with prior preventive migraine treatment failures: post hoc analysis of the DELIVER randomized clinical trial

**DOI:** 10.1111/ene.16131

**Published:** 2023-11-13

**Authors:** Messoud Ashina, Richard B. Lipton, Jessica Ailani, Jan Versijpt, Simona Sacco, Dimos D. Mitsikostas, Cecilie Laurberg Christoffersen, Bjørn Sperling, Anders Ettrup

**Affiliations:** ^1^ Danish Headache Center, Rigshospitalet Glostrup University of Copenhagen Copenhagen Denmark; ^2^ Department of Neurology Albert Einstein College of Medicine Bronx NY USA; ^3^ Department of Neurology Georgetown University Hospital Washington DC USA; ^4^ Department of Neurology Vrije Universiteit Brussel (VUB), Universitair Ziekenhuis Brussel (UZ Brussel) Brussels Belgium; ^5^ Department of Biotechnological and Applied Clinical Sciences University of L'Aquila L'Aquila Italy; ^6^ First Neurology Department, Aeginition Hospital, Medical School National and Kapodistrian University of Athens Athens Greece; ^7^ H. Lundbeck A/S Copenhagen Denmark

**Keywords:** anti‐CGRP, eptinezumab, migraine, preventive treatment

## Abstract

**Background and purpose:**

Eptinezumab reduced monthly migraine days more than placebo in the DELIVER study, a clinical trial with patients with difficult‐to‐treat migraine and prior preventive treatment failures. This post hoc analysis assesses the sustained response to eptinezumab at the population and patient level and evaluates the potential for response in initial non‐responders.

**Methods:**

Adults with chronic or episodic migraine and two to four prior preventive treatment failures were randomized to eptinezumab 100 mg, 300 mg or placebo every 12 weeks. Primary outcomes in this post hoc analysis are the proportion of patients with ≥30%, ≥50% or ≥75% reduction in monthly migraine days (i.e., migraine responder rates [MRRs]) during weeks 1–12 and weeks 13–24 and across 4‐week intervals. Secondary outcomes are maintenance and shifts in MRRs from weeks 1–12 to weeks 13–24.

**Results:**

Between weeks 1–12 and 13–24, ≥30% MRRs increased from 65.9% to 70.4% (100 mg) and from 71.0% to 74.5% (300 mg), versus 36.9% to 43.1% (placebo). The ≥50% and ≥75% MRRs were sustained or increased over the 24‐week period. The largest increase in ≥30% MRRs occurred after the second infusion with eptinezumab. The percentage of initial non‐responders (<30% MRRs during weeks 1–12) who experienced response (≥30% MRRs during weeks 13–24) to the second dose was 34.7% (100 mg) and 30.4% (300 mg) with eptinezumab versus 21.1% with placebo.

**Conclusion:**

Across MRR thresholds, most patients who responded to eptinezumab during weeks 1–12 maintained or improved response during weeks 13–24. More than one‐third of initial non‐responders became responders after their second infusion.

## Graphical abstract

In the DELIVER trial in patients with migraine and two to four prior preventive treatment failures, treatment with eptinezumab 100 mg and 300 mg resulted in high migraine responder rates compared with placebo. Most patients with response to eptinezumab during weeks 1–12 maintained response during weeks 13–24. About one‐third of initial non‐responders became responders after a second infusion with eptinezumab.

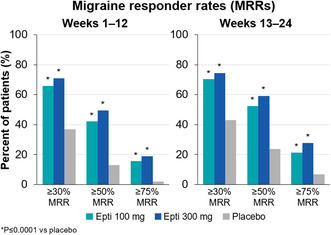

## INTRODUCTION

Migraine is a burdensome, prevalent neurological disorder [1] that reduces an individual's ability to participate in society [2]. Discontinuation of traditional oral preventive medications is common due to insufficient efficacy or poor tolerability [3]. There remains an unmet need for preventive treatment that provides robust, well‐tolerated and sustained migraine prevention that reduces monthly headache day frequency and headache‐related disability as well as improving health‐related quality of life. Several treatments targeting calcitonin gene‐related peptide (CGRP) or its receptor have been approved in recent years for the preventive treatment of migraine with the goal of reducing migraine frequency and associated burden, increasing health‐related quality of life and improving patient adherence to treatment [4, 5, 6, 7, 8].

In many countries, people suffering from migraine must fail multiple preventive treatments before they are prescribed an anti‐CGRP monoclonal antibody (i.e., eptinezumab, erenumab, fremanezumab and galcanezumab) [9, 10, 11, 12]. The European Headache Federation and the European Academy of Neurology endorse these anti‐CGRP monoclonal antibodies as third‐line preventive medications [13]. Guidance from the National Institute for Health and Care Excellence (NICE) in the United Kingdom recommends failure of at least three preventive migraine treatments prior to prescribing erenumab [10], fremanezumab [11] and galcanezumab [12], with eptinezumab guidance in development [9]. The French National Authority for Health (*Haute Autorité de Santé*), Canadian Agency for Drugs and Technologies in Health (CADTH) and the US Institute for Clinical and Economic Review recommend at least two prior preventive treatment failures [14, 15, 16, 17, 18, 19, 20].

Continued preventive treatment is contingent on the level of response experienced over 3–6 months, with assessments made after 3 months of treatment and response typically meaning the reduction in monthly migraine days (MMDs) since starting treatment [21]. CADTH recommends stopping anti‐CGRP treatment if the reduction in MMDs is <50%, although it notes that, for renewal, “some jurisdictions may want to include a reduction of at least 30% in the number of headache days per month” [16, 17, 18, 19]. In patients with chronic migraine, NICE recommends stopping treatment if response is <30% after multiple treatment cycles (vs. <50% in episodic migraine), in alignment with the position of the International Headache Society Clinical Trials Subcommittee and the Danish Medical Council, which deemed a ≥30% migraine responder rate (MRR) in chronic migraine to be clinically meaningful [22, 23, 24]. These reductions in MMDs characterized as MRRs are known as MRR thresholds.

Eptinezumab is a humanized monoclonal antibody that inhibits CGRP and is indicated for the preventive treatment of migraine in adults in the United States, Europe and other regions [25]. The efficacy and safety of eptinezumab was established in multiple large‐scale phase 3 clinical trials, which demonstrated rapid and sustained preventive effects in patients with episodic and chronic migraine [26, 27, 28]. One of these studies, DELIVER, was a phase 3b clinical study to evaluate the efficacy and safety of eptinezumab for migraine prevention in patients with two to four prior preventive treatment failures [28]. The primary data from the placebo‐controlled portion of DELIVER showed reductions in migraine frequency and severity with acceptable safety and tolerability in patients with difficult‐to‐treat migraine [28].

Given differences in prescribing and renewal guidelines for anti‐CGRP monoclonal antibodies across countries, it is important to understand the response for patients treated with these medications across a variety of thresholds. The aim here is to illuminate the expectations for the maintenance and shift in migraine responder status (i.e., 30%–49%, 50%–74% or ≥75% MRR) in patients with migraine and two to four prior treatment failures treated with eptinezumab versus placebo in the DELIVER study. The objective of these analyses was to evaluate the sustained response at both the population level and patient level during the 24‐week, placebo‐controlled portion of DELIVER. In addition, these analyses assessed the proportion of response in patients who were initially non‐responders.

## METHODS

### Study design

DELIVER was a multinational, multicenter, parallel‐group, double‐blind, randomized, placebo‐controlled phase 3b clinical study. The protocol and statistical analysis plan have been previously published, including full details of the study design, inclusion and exclusion criteria, efficacy and safety outcomes, and statistical considerations [28]. Some methodological details are briefly summarized here.

### Patients

Eligible patients included adults aged 18–75 years with onset of migraine at ≤50 years of age and with a history of migraine [29] for ≥12 months before screening. Patients had to have documented evidence of two to four prior preventive migraine treatment failures of different classes (i.e., propranolol/metoprolol, topiramate, amitriptyline, flunarizine, candesartan, valproate/divalproex and botulinum toxin A/B) in the past 10 years. Documented evidence included medical record or physician's confirmation, and at least one prior treatment failure must have been due to inadequate efficacy, tolerability reasons or contraindications. Patients with prior treatment failure with a CGRP inhibitor history or diagnosis of other headache types (e.g., chronic tension‐type headache, cluster headache etc.) or history of clinically significant cardiovascular disease were excluded.

### Treatments

Patients were randomized equally to eptinezumab 100 mg, eptinezumab 300 mg or placebo, administered by intravenous infusion at baseline (day 0) and week 12. Randomization was stratified by monthly headache days at baseline (≤14 or >14) and by country. The intravenous infusion was administered over 30 min.

To capture headache occurrence, patients completed a daily electronic diary (eDiary) with an evening report (completed daily even in the absence of headache) and a headache report (completed for each headache). Patients had four scheduled in‐person visits (screening, baseline, end of week 12 and end of week 24) and four telephone contact visits (end of weeks 4, 8, 16 and 20).

### Outcomes

The primary efficacy end‐point of DELIVER was change from baseline in the number of MMDs following the first infusion (weeks 1–12). Key secondary end‐points included the ≥50% MRR and ≥75% MRR during weeks 1–12. Other prespecified secondary end‐points included ≥50% and ≥75% MRRs following the second infusion (weeks 13–24). Exploratory end‐points analyzed here include the ≥50% and ≥75% MRRs over 4‐week intervals (i.e., weeks 1–4, 5–8, 9–12, 13–16, 17–20 and 21–24). The main outcome in this post hoc analysis was the ≥30% MRR over 4‐ and 12‐week intervals, and the secondary outcome was the shifts between migraine response thresholds from weeks 1–12 to weeks 13–24.

### Statistical analysis

Full details of sample size calculations from the DELIVER trial have been published [28]. The power was determined by simulating the testing strategy, assuming normal distributions with means and standard deviations for continuous end‐points and success rates. Briefly, with 280 patients per treatment group, the power was calculated to be at least 90% for the primary end‐point and at least 68% for individual key secondary end‐points (i.e., ≥50% and ≥75% MRRs during weeks 1–12) based on simulations. The full analysis set comprised all randomized patients who received at least one dose of study medication and had at least one valid post‐baseline 4‐week assessment of MMDs in weeks 1–12.

In this post hoc analysis, MRRs for each 4‐week interval were calculated as the change from baseline in MMDs during the given interval. MRRs across three 4‐week intervals were calculated as the average percentage change in MMDs, based on available monthly values. The treatment comparison was based on a logistic regression model, including baseline MMDs as a continuous covariate with treatment and stratification (monthly headache days at baseline ≤14 or >14) as factors. If the MMD value was missing for a given month, the responder status was derived based on the available values. MMDs were calculated by prorating if the eDiary was completed on at least 14 of the 28 days of each 4‐week period. A missed day was defined as one on which the patient did not complete the evening report or the headache report. Missing data were imputed over 4‐week periods using MMDs divided by the number of days with observations × 28 as a 1‐month score for that 4‐week period. For analyses of the shifts in response threshold, patients with a non‐missing migraine responder status for weeks 1–12 and weeks 13–24 were evaluated.

Comparisons between active treatment and placebo were based on the odds ratios of response using the likelihood ratio test. *p* values for key secondary end‐points (i.e., ≥50% and ≥75% MRRs during weeks 1–12) were based on two‐sided tests controlled for multiplicity. The DELIVER study was not designed to investigate differences between different doses of active treatment but rather between active treatment and placebo. *p* values for all other end‐points are presented with nominal *p* values and 95% confidence intervals, with no control for multiplicity; multiplicity was not controlled for beyond the end‐points included in the predefined statistical hierarchy. All statistical analyses were conducted using SAS (version 9.4 or later).

## RESULTS

### Patients

Comprehensive details of study design, patient disposition, demographics, baseline characteristics, primary and key secondary efficacy end‐points and safety/tolerability outcomes have been published previously [28]. The full analysis set comprised 890 patients (100 mg, *n* = 299; 300 mg, *n* = 293; placebo, *n* = 298), and 97.2% (865/890) completed the 24‐week placebo‐controlled treatment period. Withdrawal due to lack of efficacy comprised 0.4% (4/891) of patients. Patients with a non‐missing responder status for both weeks 1–12 and 13–24 included 287 patients who received eptinezumab 100 mg, 286 patients who received eptinezumab 300 mg and 295 patients who received placebo.

The mean (standard deviation) age of the full analysis set was 43.8 (10.6) years. A total of 800 patients (89.9%; 800/890) were female and 854 (96.0%; 854/890) were White. All but one patient had experienced at least one prior treatment failure due to lack of efficacy, 494/890 (55.5%) had at least one prior treatment failure due to safety/tolerability concerns and 28/890 (3.1%) had at least one prior treatment failure due to contraindication.

During the 28‐day baseline period, mean (standard deviation) MMDs were 13.8 (5.6) in the eptinezumab 100 mg group, 13.7 (5.4) in the eptinezumab 300 mg group and 13.9 (5.7) in the placebo group. Based on the number of migraine and headache days during the baseline period, 484/890 (54.4%) patients had episodic migraine and 405/890 (45.5%) had chronic migraine; one patient did not fit into either group after re‐calculation of baseline values.

### Migraine responder rates

**FIGURE 1:**
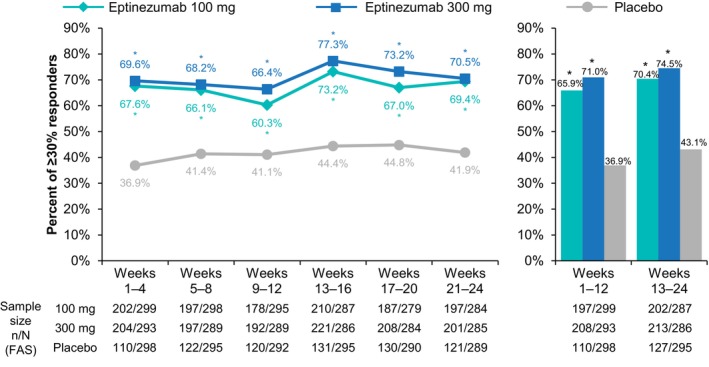
Percentage of patients achieving a ≥30% migraine responder rate over 4‐week and 12‐week intervals. *p ≤ 0.0001 vs. placebo. FAS, full analysis set.

More eptinezumab‐treated patients achieved ≥30% migraine response than did patients given placebo (Figure 1; Table S1). During the first dosing interval (weeks 1–12), 65.9% (*n* = 197/299, 100 mg; *p* < 0.0001) and 71.0% (*n* = 208/293, 300 mg; *p* < 0.0001) of patients achieved ≥30% migraine response with eptinezumab, compared with 36.9% (*n* = 110/298) with placebo. During the second dosing interval (weeks 13–24), ≥30% MRRs increased to 70.4% (*n* = 202/287, 100 mg; *p* < 0.0001) and 74.5% (*n* = 213/286, 300 mg; *p* < 0.0001) with eptinezumab and 43.1% (*n* = 127/295) with placebo. When analyzed by 4‐week intervals, the largest increase in ≥30% MRRs occurred between weeks 9–12 and weeks 13–16 with eptinezumab, representing an increase in effect with a second infusion of active treatment. An exploratory analysis identified that this increase in effect was observed in the episodic migraine population, and maintenance of the increased effect was observed in the chronic migraine population (Table S2). Although the recommended threshold for clinical response is ≥50% MRR for episodic migraine, ≥30% MRR was analyzed here for comparison to chronic migraine.

**FIGURE 2:**
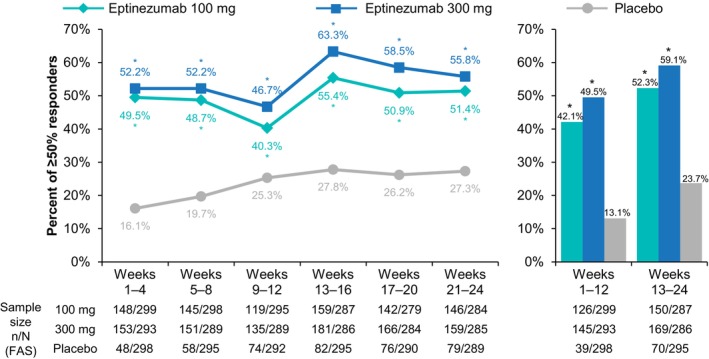
Percentage of patients achieving a ≥50% migraine responder rate over 4‐week and 12‐week intervals. *p ≤ 0.0001 vs. placebo. FAS, full analysis set.

**FIGURE 3:**
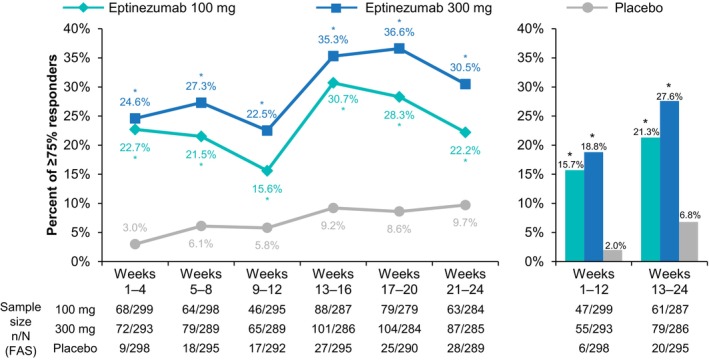
Percentage of patients achieving a ≥75% migraine responder rate over 4‐week and 12‐week intervals. *p ≤ 0.0001 vs. placebo. FAS, full analysis set.

The ≥50% MRRs (Figure 2; Table S3) and ≥75% MRRs (Figure 3; Table S4) followed a similar pattern to ≥30% MRRs, wherein eptinezumab treatment resulted in higher rates of response than placebo in each 4‐ and 12‐week interval, with the largest increase in MRR occurring in the 4 weeks following the second infusion of eptinezumab (i.e., between weeks 9–12 and 13–16) for both the 100 mg and 300 mg doses.

### Maintained and shifts in migraine response

**FIGURE 4:**
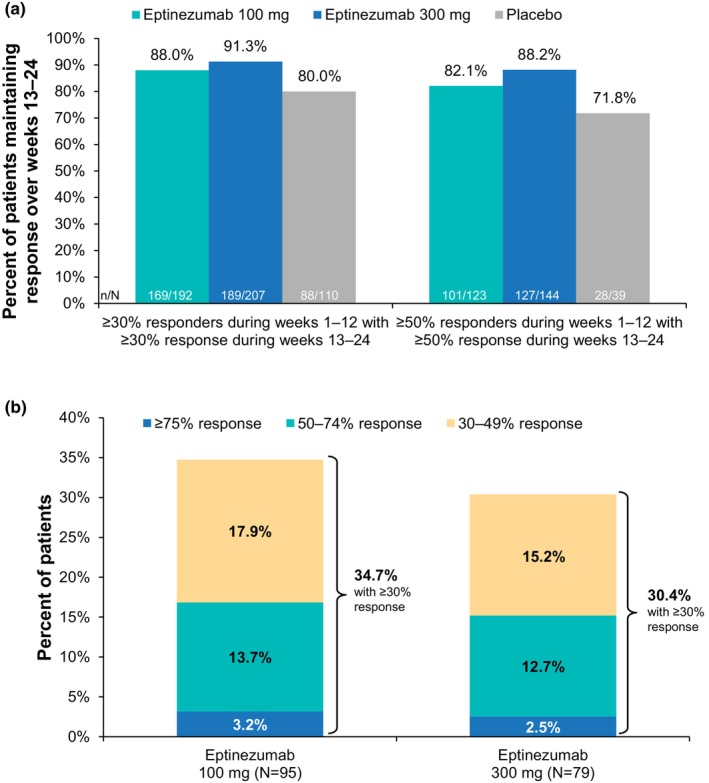
(a) Percentage of patients maintaining response between weeks 1–12 and 13–24 and (b) shift in migraine response in patients with <30% response during weeks 1–12.

Across eptinezumab treatment groups, ≥30% and ≥50% MRRs during weeks 1–12 were maintained during weeks 13–24 by >80% of patients (Figure 4a). Lower maintenance was observed with placebo, except for ≥75% migraine response; however, only six patients who received placebo achieved ≥75% migraine response during weeks 1–12. The percentage of patients who consistently had <30% migraine response across both dosing intervals was 21.6% (*n* = 62/287, 100 mg) and 19.2% (*n* = 55/286, 300 mg) with eptinezumab compared with 49.5% (*n* = 146/295) with placebo.

The shifts in responder threshold (i.e., <30%, 30%–49%, 50%–74% and ≥75%) during weeks 13–24 in the subgroup of patients with <30% migraine response during weeks 1–12 are shown in Figure 4b (and Figure S1). The percentage of patients with <30% migraine response during weeks 1–12 who had ≥30% response during weeks 13–24 was 34.7% (*n* = 33/95, 100 mg) and 30.4% (*n* = 24/79, 300 mg) with eptinezumab compared with 21.1% (*n* = 39/185) with placebo. Similar results were observed for ≥50% migraine response during weeks 13–24, with 16.8% (*n* = 16/95, 100 mg) and 15.2% (*n* = 12/79, 300 mg) of active treated patients reporting a ≥50% migraine response versus 6.5% (*n* = 12/185) of patients treated with placebo.

Of patients with 30%–49% migraine response during weeks 1–12, 47.8% (*n* = 33/69, 100 mg) and 47.6% (*n* = 30/63, 300 mg) of eptinezumab‐treated patients had ≥50% response during weeks 13–24 compared with 42.3% (*n* = 30/71) of patients given placebo (Figure S2). Approximately 80% of eptinezumab‐treated patients with 50%–74% migraine response during weeks 1–12 maintained or improved responder status during weeks 13–24 compared with about 70% of patients given placebo (Figure S3). Migraine response in the ≥75% MRR threshold was maintained by >60% of patients (Figure S4). For both doses of eptinezumab, the percentage of patients who achieved ≥30%, ≥50% and ≥75% MRRs during weeks 1–12 but became non‐responders during weeks 13–24 was 4.6% (*n* = 41/890).

## DISCUSSION

Given the differences in prescribing guidelines and criteria for treatment initiation and discontinuation for anti‐CGRP monoclonal antibodies across countries, it is important to understand the potential trajectory of response for patients treated with these medications across a variety of thresholds. The data presented here may guide clinicians to continue treatment with eptinezumab for at least two infusions before making dosage change evaluations or discontinuing treatment. The current analysis illuminates the potential expectations for the maintenance and shift in migraine responder status in patients with migraine and multiple prior treatment failures treated with eptinezumab versus placebo in the DELIVER study. The results highlight that significantly more patients treated with eptinezumab than with placebo will experience migraine response and that a substantial proportion of eptinezumab‐treated patients will experience a maintained or improved migraine response over 24 weeks of treatment. Additionally, approximately one‐third of eptinezumab‐treated patients with insufficient migraine response during the first dosing interval (weeks 1–12) responded after a second infusion (weeks 13–24) in both the 100 mg and 300 mg treatment groups.

The ≥50% and ≥75% MRRs during weeks 1–12 and 13–24 in the eptinezumab groups were consistent with data from the pivotal PROMISE studies in episodic [30] and chronic migraine [31], but placebo MRRs in the PROMISE studies were higher than observed in this study where patients have experienced two to four prior preventive treatment failures. A post hoc analysis of the PROMISE studies found that 37.6% (100 mg), 36.3% (300 mg) and 33.9% (placebo) of patients with <50% migraine response during weeks 1–12 in PROMISE‐1 experienced ≥50% response during weeks 13–24; in PROMISE‐2, these percentages were 28.7% (100 mg), 29.0% (300 mg) and 18.5% (placebo) [32]. Thus, across three trials of eptinezumab in different migraine populations, approximately a third of eptinezumab‐treated patients who did not experience response during the first infusion experienced migraine response after a second infusion, highlighting the importance of a series of at least two treatments. Whilst eptinezumab does has a rapid onset of action [33, 34], related to its intravenous route of administration, some patients, due to the natural fluctuation of disease [35], may require additional time with preventive treatment to gain the full benefits.

DELIVER comprised patients with episodic or chronic migraine with documented evidence of two to four prior preventive treatment failures. Patients with multiple treatment failures could be considered to have resistant or refractory migraine [21]. Resistant migraine is defined as at least three failures of different preventive migraine medication classes with at least eight debilitating headache days per month for at least three consecutive months; preventive treatment refractory migraine is defined as failure of all available preventive migraine medication classes with at least eight debilitating headache days per month for at least six consecutive months [21]. Patients with resistant or refractory migraine experience a negative impact on quality of life and personal, professional and social activities, as well as increased healthcare costs [2], and patients can feel hopeless about their disease state and remission. Resistant or refractory migraine may be more difficult to treat, and although the demographic was not captured in this study a proportion of the DELIVER population may have met resistant or refractory migraine criteria. The ≥30% MRR threshold has been deemed clinically meaningful by the International Headache Society Clinical Trials Subcommittee [22, 23] and is a treatment‐renewal guideline per NICE and CADTH [10, 11, 12, 16, 17, 18]. Beyond guidelines, whether treatment is providing meaningful benefit to the patient should be amongst the items discussed between clinicians and their patients when determining next steps with treatment.

Although the observed placebo response over 24 weeks in the DELIVER study was lower than that observed in the PROMISE studies [30, 31], 2% of DELIVER patients given placebo experienced ≥75% migraine response, 13% experienced ≥50% migraine response and 37% experienced ≥30% migraine response, with each MRR slightly increasing after a second infusion. The largest 4‐week increases in MRRs with placebo tended to occur within dosing intervals (≥30%, 4.5 percentage‐point increase from weeks 1–4 to 5–8; ≥50%, 5.6 percentage‐point between weeks 5–8 and 9–12; ≥75%, 3.4 percentage‐point between weeks 9–12 and 13–16), whereas the largest increase in MRR with eptinezumab occurred immediately following the second infusion (i.e., weeks 13–16). With eptinezumab, the largest percentage‐point differences always occurred between weeks 9–12 and 13–16 and ranged from 10.9 to 16.6 percentage‐points. The placebo response is unlikely to be biased by dropout rate, which was less than 3% of patients overall and less than 0.5% due to lack of efficacy [28]. The percentage‐point differences with eptinezumab may be related to a slight end‐of‐dose deterioration effect that is not observed with placebo; however, further analysis of longer‐term data is needed to assess if the end‐of‐dose effect remains beyond the two infusions in the placebo‐controlled period. These differences also underscore the benefit of a second infusion with eptinezumab that is not observed with placebo.

### Study limitations

DELIVER may have limited generalizability to the general migraine population, given that the study population was mainly female and White and MRRs were not analyzed by sex or race subgroups. Individuals with previous anti‐CGRP therapy failures were excluded from participation, as were those with cardiovascular disease or certain pain syndromes; thus, the findings may have limited generalizability in patients with these or other excluded conditions.

Sustained dosing of eptinezumab 100 mg was associated with incremental improvements; however, the DELIVER study did not assess dose escalation so comparative effectiveness remains to be explored. It may be of interest to clinicians and payers to determine when dosage should increase from 100 mg to 300 mg, although no data are currently available to predict which patients will respond better to the higher dose of eptinezumab [36]. Additionally, intravenous infusion with CGRP antibodies may lead to a higher placebo response [37]. The placebo response here increased over the course of the 6‐month treatment period, which could be due to the nature and context of treatment administration and fluctuation of disease.

## CONCLUSION

In the DELIVER study, ≥30%, ≥50% and ≥75% MRRs were higher with eptinezumab than with placebo, and the MRRs over time supported a beneficial effect of a second infusion of eptinezumab. While many healthcare providers traditionally use ≥50% migraine response as the threshold for defining clinically meaningful benefit, these data inform clinicians on rates of patients fulfilling the threshold for additional dosing in patients with migraine and prior preventive treatment failures. Further, more than one in three patients who did not achieve migraine response with eptinezumab during the first dosing interval did achieve response following the second infusion; thus, these data support that two infusions of eptinezumab may be needed before evaluating treatment outcome.

## AUTHOR CONTRIBUTIONS

Messoud Ashina: Conceptualization (equal), investigation (equal), writing—reviewing and editing (equal). Richard B. Lipton: Writing—original draft preparation (equal), writing—reviewing and editing (equal). Jessica Ailani: Conceptualization (equal), writing—original draft preparation (equal), writing—reviewing and editing (equal). Jan Versijpt: Conceptualization (equal), investigation (equal), writing—original draft preparation (equal), writing—reviewing and editing (equal). Simona Sacco: Conceptualization (equal), writing—original draft preparation (equal), writing—reviewing and editing (equal). Dimos D. Mitsikostas: Writing—original draft preparation (equal), writing—reviewing and editing (equal). Cecilie Laurberg Christoffersen: Conceptualization (equal), funding acquisition (equal), data curation (lead), formal analysis (lead), methodology (equal), software (equal), validation (equal), writing—original draft preparation (equal), writing—reviewing and editing (equal). Bjørn Sperling: Conceptualization (equal), funding acquisition (equal), writing—original draft preparation (equal), writing—reviewing and editing (equal). Anders Ettrup: Conceptualization (equal), funding acquisition (equal), writing—original draft preparation (equal), writing—reviewing and editing (equal).

## FUNDING INFORMATION

The clinical trial and publication were funded by H. Lundbeck A/S, Copenhagen, Denmark.

## CONFLICT OF INTEREST STATEMENT

MA has received personal fees from AbbVie, Amgen, Eli Lilly, GlaxoSmithKline, Lundbeck, Novartis, Pfizer and Teva Pharmaceuticals during the conduct of the study; has received research support from Lundbeck Foundation, Novo Nordisk Foundation and Novartis; and has served as associate editor of *Cephalalgia*, associate editor of *The Journal of Headache and Pain* and associate editor of *Brain*. RBL has been a consultant, advisory board member and/or has received honoraria from Allergan/AbbVie, American Academy of Neurology, American Headache Society, Amgen, Biohaven Pharmaceuticals, BioVision, Boston Scientific, Dr Reddy's Laboratories, electroCore, Eli Lilly, eNeura Therapeutics, GlaxoSmithKline, Lundbeck Seattle BioPharmaceuticals, Merck, Pernix, Pfizer, Supernus, Teva, Trigemina, Vector and Vedanta; has received compensation from eNeura and Biohaven Pharmaceuticals; has stock or stock options in Biohaven Pharmaceuticals; and has received research support from Amgen, Migraine Research Foundation and National Headache Foundation. JA has received research support from Allergan/AbbVie, Biohaven, Eli Lilly, Satsuma and Zosano; consulting fees from Aeon, Allergen/AbbVie, Amgen, Axsome, Biodelivery Sciences International, Biohaven, Eli Lilly, GlaxoSmithKline, Impel, Linpharma, Lundbeck, Miravo, Nesos, Pfizer, Satsuma, Teva and Theranica. JV has received consulting fees from and has participated on advisory boards for Allergan/AbbVie, Eli Lilly, Lundbeck, Novartis and Teva; has received payment or honoraria for presentations from Eli Lilly and Teva; support for attending meetings or travel from Allergan/AbbVie and Teva; is a board member of the European Headache Federation; and is the president of the Belgian Headache Society. SS has received grants or contracts from Novartis and Uriach; has received consulting fees and payment or honoraria for presentations from Abbot, Allergan/AbbVie, AstraZeneca, Eli Lilly, Lundbeck, Novartis, NovoNordisk and Pfizer; support for attending meetings or travel from Eli Lilly, Lundbeck, Novartis and Teva; equipment or services from Allergan/AbbVie and NovoNordisk; is the second vice‐president for European Headache Federation; and is the president‐elect for European Stroke Organization. DDM has received consulting fees from Eli Lilly, Lundbeck, Novartis and Teva; payment or honoraria for presentations from Allergan/AbbVie, Eli Lilly, Lundbeck, Novartis and Teva; support for attending meetings or travel from Allergen/AbbVie, Eli Lilly, Genesis, Lundbeck, Novartis and Teva; is the co‐chair of Headache Panel at European Academy of Neurology; and is the president of Hellenic Headache Society. CLC, BS and AE are full‐time employees of H. Lundbeck A/S or one of its subsidiary companies. BS owns stock in Lundbeck or one of its subsidiary companies.

## ETHICS STATEMENT

The study was conducted in accordance with standards of Good Clinical Practice as defined by the International Conference on Harmonisation and all applicable federal and local regulations. The local review board or a central institutional review board/ethics committee approved all study documentation at each of the 96 study sites across Europe and the United States. All patients provided written informed consent prior to study participation. DELIVER is registered on ClinicalTrials.gov (NCT04418765) and EudraCT (2019–004497‐25).

## Supporting information


Appendix S1

## Data Availability

In accordance with EFPIA's and PhRMA's “Principles for Responsible Clinical Trial Data Sharing” guidelines, Lundbeck is committed to responsible sharing of clinical trial data in a manner that is consistent with safeguarding the privacy of patients, respecting the integrity of national regulatory systems, and protecting the intellectual property of the sponsor. The protection of intellectual property ensures continued research and innovation in the pharmaceutical industry. Deidentified data are available to those whose request has been reviewed and approved through an application submitted to https://www.lundbeck.com/global/our‐science/clinical‐data‐sharing.
