# Supplementary material for: Responder rates with eptinezumab over 24 weeks in patients with prior preventive migraine treatment failures: post hoc analysis of the DELIVER randomized clinical trial
Source: Eur J Neurol. 2023 Nov 13;31(2):e16131. doi: 10.1111/ene.16131 (PMC11235785; doi:10.1111/ene.16131)

SUPPLEMENTAL MATERIAL

Supplemental Table 1. Patients achieving ≥30% migraine responder rate over 4-week and 12-week intervals

	Eptinezumab 100 mg				Eptinezumab 300 mg				Placebo
**Time Period**	**n/N (%)**	**Δ Placebo**	**OR (95% CI)**	***P*-Value**	**n/N (%)**	**Δ Placebo**	**OR (95% CI)**	***P*-Value**	**n/N (%)**
**Weeks 1–4**	202/299 (67.6)	30.6	3.66 (2.61, 5.17)	<0.0001	204/293 (69.6)	32.7	3.98 (2.83, 5.65)	<0.0001	110/298 (36.9)
**Weeks 5–8**	197/298 (66.1)	24.8	2.80 (2.00, 3.92)	<0.0001	197/289 (68.2)	26.8	3.04 (2.17, 4.29)	<0.0001	122/295 (41.4)
**Weeks 9–12**	178/295 (60.3)	19.2	2.20 (1.58, 3.07)	<0.0001	192/289 (66.4)	25.3	2.85 (2.04, 4.02)	<0.0001	120/292 (41.1)
**Weeks 13–16**	210/287 (73.2)	28.8	3.48 (2.46, 4.97)	<0.0001	221/286 (77.3)	32.9	4.37 (3.05, 6.32)	<0.0001	131/295 (44.4)
**Weeks 17–20**	187/279 (67.0)	22.2	2.51 (1.79, 3.54)	<0.0001	208/284 (73.2)	28.4	3.37 (2.38, 4.81)	<0.0001	130/290 (44.8)
**Weeks 21–24**	197/284 (69.4)	27.5	3.17 (2.25,4.48)	<0.0001	201/285 (70.5)	28.7	3.35 (2.38, 4.76)	<0.0001	121/289 (41.9)
**Weeks 1–12**	197/299 (65.9)	29.0	3.35 (2.40,4.72)	<0.0001	208/293 (71.0)	34.1	4.21 (2.99, 5.98)	<0.0001	110/298 (36.9)
**Weeks 13–24**	202/287 (70.4)	27.3	3.18 (2.26,4.50)	<0.0001	213/286 (74.5)	31.4	3.92 (2.76, 5.61)	<0.0001	127/295 (43.1)

Δ, difference from; OR, odds ratio; CI, confidence interval.

Supplemental Table 2. Patients with EM or CM achieving a ≥30% migraine responder rate over 4-week and 12-week intervals

Episodic Migraine							
	Eptinezumab 100 mg			Eptinezumab 300 mg			Placebo
**Time Period**	**n/N (%)**	**OR (95% CI)**	***P*-Value**	**n/N (%)**	**OR (95% CI)**	***P*-Value**	**n/N (%)**
**Weeks 1–4**	117/162 (72.2)	3.97 (2.51, 6.36)	<0.0001	117/158 (74.1)	4.35 (2.72, 7.04)	<0.0001	65/164 (39.6)
**Weeks 5–8**	111/161 (68.9)	2.78 (1.77, 4.41)	<0.0001	109/158 (69.0)	2.78 (1.77, 4.42)	<0.0001	72/162 (44.4)
**Weeks 9–12**	100/160 (62.5)	2.00 (1.28, 3.13)	0.0023	112/158 (70.9)	2.90 (1.83, 4.65)	<0.0001	73/160 (45.6)
**Weeks 13–16**	126/156 (80.8)	4.57 (2.79, 7.66)	<0.0001	126/156 (80.8)	4.54 (2.77, 7.60)	<0.0001	79/164 (48.2)
**Weeks 17–20**	101/150 (67.3)	2.45 (1.55, 3.91)	0.0001	121/156 (77.6)	4.11 (2.54, 6.75)	<0.0001	74/162 (45.7)
**Weeks 21–24**	112/154 (72.7)	3.48 (2.18, 5.64)	<0.0001	118/155 (76.1)	4.13 (2.56, 6.77)	<0.0001	71/164 (43.8)
**Weeks 1–12**	110/162 (67.9)	3.16 (2.01, 5.01)	<0.0001	116/158 (73.4)	4.11 (2.58, 6.64)	<0.0001	66/164 (40.2)
**Weeks 13–24**	117/156 (75.0)	3.53 (2.20, 5.73)	<0.0001	123/156 (78.8)	4.35 (2.68, 7.20)	<0.0001	76/164 (46.3)

Chronic Migraine							
	Eptinezumab 100 mg			Eptinezumab 300 mg			Placebo
**Time Period**	**n/N (%)**	**OR (95% CI)**	***P*-Value**	**n/N (%)**	**OR (95% CI)**	***P*-Value**	**n/N (%)**
**Weeks 1–4**	85/137 (62.0)	3.32 (2.01, 5.55)	<0.0001	86/134 (64.2)	3.55 (2.14, 5.96)	<0.0001	45/134 (33.6)
**Weeks 5–8**	86/137 (62.8)	2.82 (1.72, 4.68)	<0.0001	87/130 (66.9)	3.32 (2.00, 5.59)	<0.0001	50/133 (37.6)
**Weeks 9–12**	78/135 (57.8)	2.48 (1.51, 4.12)	0.0003	79/130 (60.8)	2.79 (1.69, 4.67)	<0.0001	47/132 (35.6)
**Weeks 13–16**	84/131 (64.1)	2.69 (1.63, 4.48)	<0.0001	94/129 (72.9)	4.04 (2.41, 6.91)	<0.0001	52/131 (39.7)
**Weeks 17–20**	86/129 (66.7)	2.54 (1.53, 4.27)	0.0003	87/127 (68.5)	2.77 (1.66, 4.68)	<0.0001	56/128 (43.8)
**Weeks 21–24**	85/130 (65.4)	2.89 (1.75, 4.85)	<0.0001	83/129 (64.3)	2.76 (1.67, 4.62)	<0.0001	50/127 (39.4)
**Weeks 1–12**	87/137 (63.5)	3.64 (2.20, 6.10)	<0.0001	91/134 (67.9)	4.34 (2.60, 7.35)	<0.0001	44/134 (32.8)
**Weeks 13–24**	85/131 (64.9)	2.88 (1.74, 4.82)	<0.0001	90/129 (69.8)	3.59 (2.15, 6.09)	<0.0001	51/131 (38.9)

EM, episodic migraine; CM, chronic migraine; OR, odds ratio; CI, confidence interval.

Supplemental Table 3. Patients achieving ≥50% migraine responder rate over 4-week and 12-week intervals

	Eptinezumab 100 mg				Eptinezumab 300 mg				Placebo
**Time Period**	**n/N (%)**	**Δ Placebo**	**OR (95% CI)**	***P*-Value**	**n/N (%)**	**Δ Placebo**	**OR (95% CI)**	***P*-Value**	**n/N (%)**
**Weeks 1–4**	148/299 (49.5)	33.4	5.28 (3.61, 7.85)	<0.0001	153/293 (52.2)	36.1	5.86 (4.00, 8.72)	<0.0001	48/298 (16.1)
**Weeks 5–8**	145/298 (48.7)	29.0	4.04 (2.80, 5.89)	<0.0001	151/289 (52.2)	32.6	4.60 (3.18, 6.72)	<0.0001	58/295 (19.7)
**Weeks 9–12**	119/295 (40.3)	15.0	2.00 (1.41, 2.85)	0.0001	135/289 (46.7)	21.4	2.59 (1.83, 3.70)	<0.0001	74/292 (25.3)
**Weeks 13–16**	159/287 (55.4)	27.6	3.28 (2.32, 4.66)	<0.0001	181/286 (63.3)	35.5	4.59 (3.23, 6.56)	<0.0001	82/295 (27.8)
**Weeks 17–20**	142/279 (50.9)	24.7	2.96 (2.08, 4.24)	<0.0001	166/284 (58.5)	32.2	4.02 (2.83, 5.76)	<0.0001	76/290 (26.2)
**Weeks 21–24**	146/284 (51.4)	24.1	2.83 (2.00, 4.02)	<0.0001	159/285 (55.8)	28.5	3.38 (2.39, 4.82)	<0.0001	79/289 (27.3)
**Weeks 1–12**	126/299 (42.1)	29.1	4.91 (3.29, 7.47)	<0.0001	145/293 (49.5)	36.4	6.58 (4.41, 10.01)	<0.0001	39/298 (13.1)
**Weeks 13–24**	150/287 (52.3)	28.5	3.56 (2.50, 5.10)	<0.0001	169/286 (59.1)	35.4	4.69 (3.29, 6.75)	<0.0001	70/295 (23.7)

Δ, difference from; OR, odds ratio; CI, confidence interval.

Supplemental Table 4. Patients achieving ≥75% migraine responder rate over 4-week and 12-week intervals

	Eptinezumab 100 mg				Eptinezumab 300 mg				Placebo
**Time Period**	**n/N (%)**	**Δ Placebo**	**OR (95% CI)**	***P*-Value**	**n/N (%)**	**Δ Placebo**	**OR (95% CI)**	***P*-Value**	**n/N (%)**
**Weeks 1–4**	68/299 (22.7)	19.7	9.87 (5.04, 21.69)	<0.0001	72/293 (24.6)	21.6	10.94 (5.60, 24.03)	<0.0001	9/298 (3.0)
**Weeks 5–8**	64/298 (21.5)	15.4	4.26 (2.50, 7.61)	<0.0001	79/289 (27.3)	21.2	5.84 (3.46, 10.36)	<0.0001	18/295 (6.1)
**Weeks 9–12**	46/295 (15.6)	9.8	2.98 (1.70, 5.48)	0.0001	65/289 (22.5)	16.7	4.70 (2.74, 8.50)	<0.0001	17/292 (5.8)
**Weeks 13–16**	88/287 (30.7)	21.5	4.48 (2.83, 7.29)	<0.0001	101/286 (35.3)	26.2	5.54 (3.52, 8.99)	<0.0001	27/295 (9.2)
**Weeks 17–20**	79/279 (28.3)	19.7	4.29 (2.66, 7.11)	<0.0001	104/284 (36.6)	28.0	6.31 (3.96, 10.38)	<0.0001	25/290 (8.6)
**Weeks 21–24**	63/284 (22.2)	12.5	2.68 (1.67, 4.40)	<0.0001	87/285 (30.5)	20.8	4.15 (2.64, 6.72)	<0.0001	28/289 (9.7)
**Weeks 1–12**	47/299 (15.7)	13.7	9.19 (4.16, 24.35)	<0.0001	55/293 (18.8)	16.8	11.43 (5.22, 30.15)	<0.0001	6/298 (2.0)
**Weeks 13–24**	61/287 (21.3)	14.5	3.75 (2.23, 6.55)	<0.0001	79/286 (27.6)	20.8	5.32 (3.20, 9.20)	<0.0001	20/295 (6.8)

Δ, difference from; OR, odds ratio; CI, confidence interval.

Supplemental Figure 1. Shift in MRRs during Weeks 13–24 in patients with <30% migraine response during Weeks 1–12 (initial non-responders)

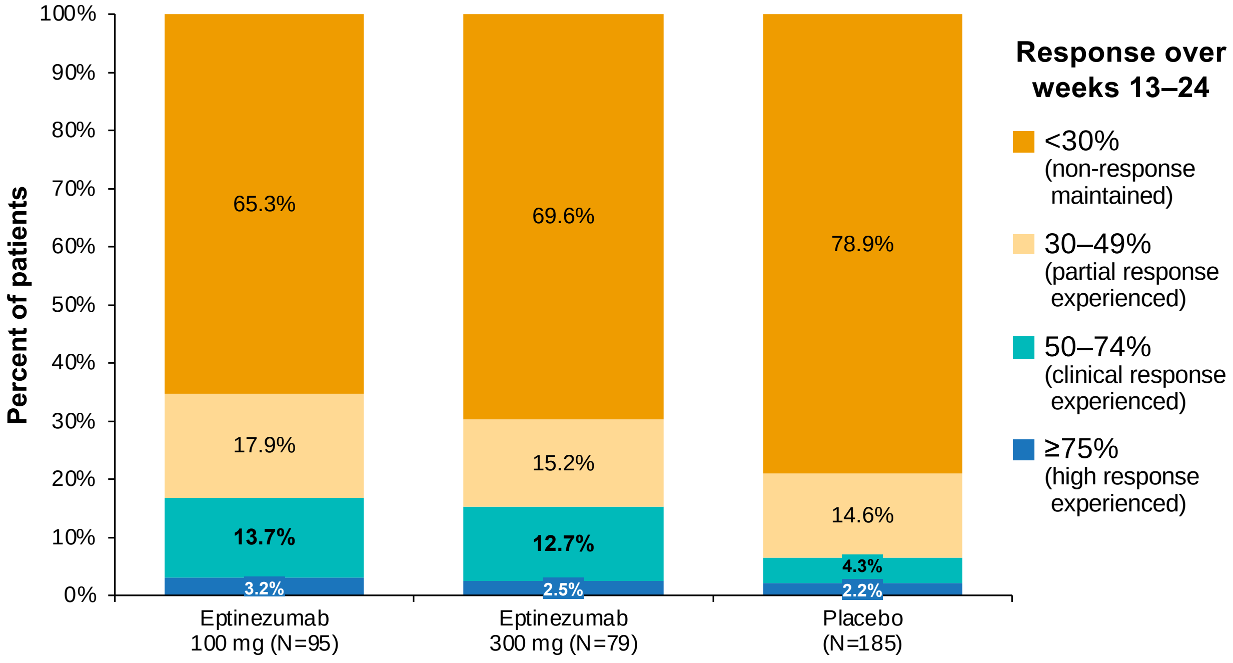

Supplemental Figure 2. Shift in MRRs during Weeks 13–24 in patients with 30–49% migraine response during Weeks 1–12 (initial non-responders)

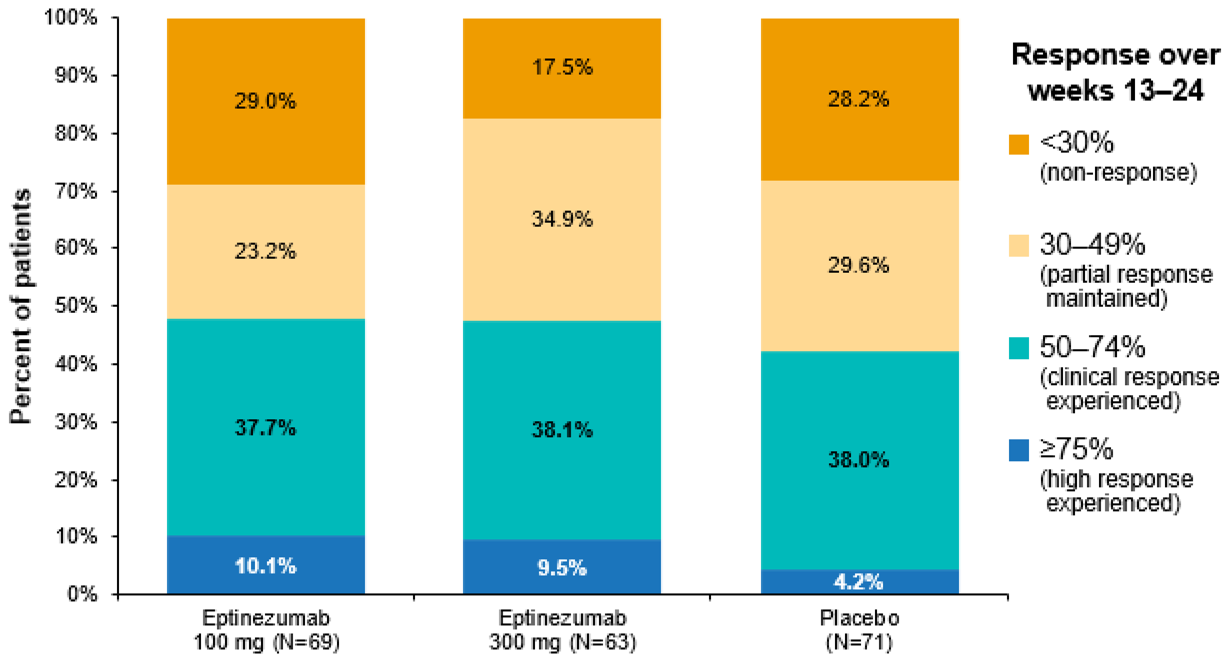

Supplemental Figure 3. Shift in MRRs during Weeks 13–24 in patients with 50–74% migraine response during Weeks 1–12 (initial non-responders)

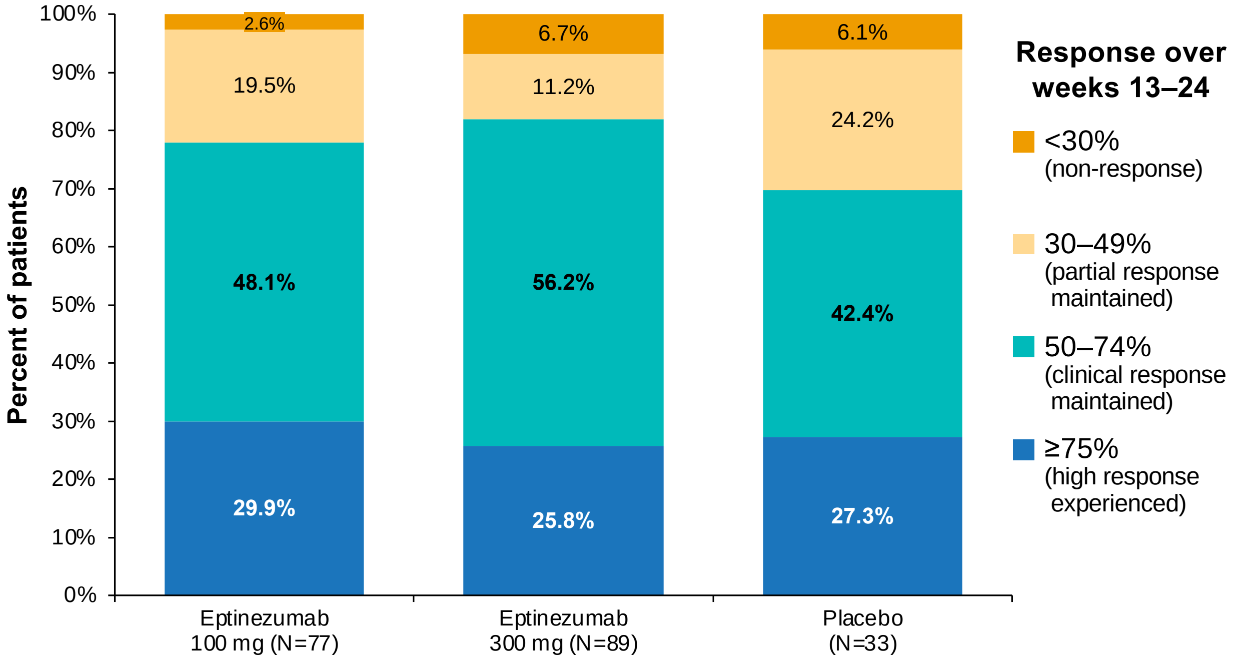

Supplemental Figure 4. Shift in MRRs during Weeks 13–24 in patients with ≥75% migraine response during Weeks 1–12 (initial non-responders)

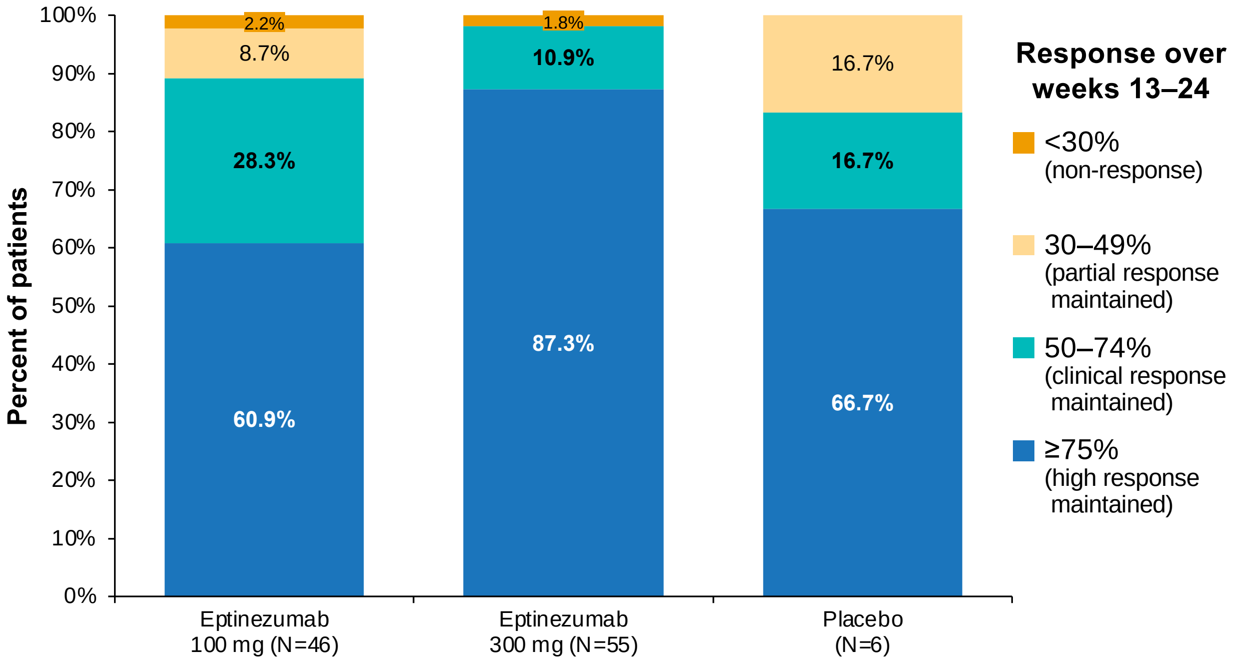

Supplement: Supplementary file 1 — Appendix S1 [file ENE-31-e16131-s001.pdf]
